# Drinking Water

**Published:** 1882-01

**Authors:** 


### DRINKING WATER.

EVEN pure cold water may be drunk too freely in summer-time. Persons who are in feeble health or suffer from the effects of summer diseases, will derive great advantage from swallowing bits of ice whole, after craunching them with their teeth, instead of taking large draughts of ice-water, which often have the effect to increase the thirst; this is not the case if ice is eaten.

A person who drinks water largely in the early part of a summer's day, will be more troubled with thirst during the remainder of the day than if these cravings had been resisted for a few hours.

The more water a man drinks in summer, the more he perspires, and after a certain point, perspiration becomes debilitating, and is then a cause of disease.

When persons are feverish and thirsty beyond what is natural, indicated in some cases by a metallic taste in the mouth, especially after drinking water, or by a whitish appearance of the greater part of the surface of the tongue, one of the best "coolers," internal or external, is to take a lemon, cut off the top, sprinkle over it some loaf sugar, working it downward into the lemon with the spoon, and then suck it slowly, squeezing the lemon and adding more sugar as the acidity increases from being brought up from a lower point. Invalids with feverishness may take two or three lemons a day in this manner with the most marked benefit, manifested by a sense of coolness, comfort, and invigoration. A lemon or two thus taken at "tea-time," as an entire substitute for the ordinary "supper" of summer, would give many a man a comfortable night's sleep and an awaking of rest and invigoration, with an appetite for breakfast, to which they are strangers who will have their cup of tea or supper of "relish" and "cake" and berries or peaches and cream.

The lemon thus eaten was the great physical solace of General Jackson in his last illness, which was consumption combined with dropsy. It loosened the cough and relieved him of much of that annoying hacking and hemming which attends diseases of the throat and lungs, many times more efficient, speedy, and safe than any lozenge, or "Trochee" ever swallowed.

We are daily in receipt of letters from persons who claim to have been cured of piles and constipation by the use of Nealton's Suppository, and who request us to recommend the remedy, which we do cheerfully. See advertisement.

**SLEEPING ROOMS.**—The air which passes out of the lungs is wholly innutritious. If re-breathed without any admixture of other air, it would induce instant suffocation. It contains a large amount of carbonic acid gas. This gas is condensed by cold, and falls to the floor; heat carries it to the ceiling; hence the practical fact, that in warm weather, those who sleep on the floor, breathe the purest air; while in very cold weather, the higher one sleeps above the floor, the better is the atmosphere. Hence, in a warm room, sleep as near the floor as possible; in a cold room, the higher the bed is, the better. A striking illustration of one branch of the statement is found in Dr. Hall's new book on SLEEP. When the jail-fever was raging in England, it was the custom to hand the food and water to the prisoners through a hole in the floor above them. A case is mentioned where the jailer and his wife died in one night, in consequence of the effluvia of the prisoners' cells below; while the prisoners themselves continued to live, showing conclusively the concentrated malignity of the air at the ceiling, as compared with that on the floor. The same principle has an illustration in the narration in the same pages, of the terrible incidents in connection with the "Black Hole of Calcutta," where it was speedily noticed that relief was given by sitting down on the floor. From these statements, it is clear, that it is better to have a fire in the fireplace in a close room in winter than to have no fire; and for two philosophical reasons—the fire rarefies the carbonic acid gas, and compels it to seek the ceiling; besides, it creates a draft up the chimney, thus causing cold air to come in more copiously

**SLEEPING POSITION.**—The food passes from the stomach at the right side, hence its passage is facilitated by going to sleep on the right side. Water and other fluids flow equably on a level, and it requires less power to propel them on a level, than upwards. The heart propels the blood to every part of the body at each successive beat, and it is easy to see that if the body is in a horizontal position the blood will be sent to the various parts of the system with greater ease, with less expenditure of power, and more perfectly than could possibly be done if one portion of the body were elevated above a horizontal line. On the other hand, if one portion of the body is too low, the blood does not return as readily as it is carried thither; hence, there is an accumulation and distention, and pain soon follows. If a person goes to sleep with the head but a very little lower than the body, he will either soon waken up, or will die with apoplexy before the morning, simply because the blood could not get back from the brain as fast as it was carried to it. If a person lays himself down on a level floor for sleep, a portion of the head, at least, is lower than the heart, and discomfort is soon induced; hence, very properly, the world over, the head is elevated during sleep. The savage uses a log of wood or a bunch of leaves; the civilized a pillow; and if this pillow is too thick, raising the head too high, there is not blood enough carried to the brain, and as the brain is nourished, renewed, and invigorated by the nutriment it receives from the blood during sleep, it is not fed sufficiently, and the result is unquiet sleep during the night, and a waking up in weariness, without refreshment, to be followed by a day of drowsiness, discomfort, and general inactivity of both mind and body. The healthful mean is a pillow, which by the pressure of the head keeps it about four inches above the level of the bed or mattress; nor should the pillow be so soft as to allow the head to be buried in it, and excite perspiration, endangering ear-ache or cold in the head, on turning over. The pillow should be hard enough to prevent the head sinking more than about three inches.

**EDUCATION** is literally "a leading from;" it may be from ignorance to knowledge; from vice to virtue. This is education in a good sense. But we may be educated, "led from" virtue to vice; from truth to error; so that in its largest acceptation, education is the being "led from" an old state or habit of thought, feeling, and action, into some other state of thought, feeling, and action. But we may educate the body as well as the mind and heart; we may educate it from a sickly, diseased condition into robust health and manly vigor; we may also bring it from these down to rottenness and imbecility. A true and comprehensive education, in reference especially to our children, is that which leads out the bodily powers to greater strength; the mental to greater activities; the moral to greater purity, beneficence, and nobility—all these should be done at the same time, although in different degrees.

